# Automated titanium fastener vs. hand-tied knots for prosthesis fixation in infective endocarditis

**DOI:** 10.3389/fcvm.2024.1363336

**Published:** 2024-01-23

**Authors:** Amila Kahrovic, Philipp Angleitner, Harald Herkner, Paul Werner, Thomas Poschner, Leila Alajbegovic, Alfred Kocher, Marek Ehrlich, Günther Laufer, Martin Andreas

**Affiliations:** ^1^ Department of Cardiac Surgery, Medical University of Vienna, Vienna, Austria; ^2^ Department of Emergency Medicine, Medical University of Vienna, Vienna, Austria

**Keywords:** infective endocarditis, automated titanium fastener, hand-tied knots, suture-securing techniques, prosthesis fixation

## Abstract

**Objectives:**

To date, there is no evidence regarding the safety of automated titanium fastener compared with hand-tied knots for prosthesis fixation in infective endocarditis.

**Methods:**

Between January 2016 and December 2022, a total of 220 patients requiring surgery for infective endocarditis were included in this retrospective analysis. The primary study endpoint was re-endocarditis during follow-up. The secondary study endpoints included stroke onset, all-cause mortality, and a composite outcome of either re-endocarditis, stroke, or all-cause mortality during follow-up.

**Results:**

Suture-securing with an automated titanium fastener was performed in 114 (51.8%) patients, whereas the conventional technique of hand knot-tying was used in 106 (48.2%) patients. The risk of re-endocarditis was significantly lower in the automated titanium fastener group, as shown in a multivariable proportional competing risk regression model (adjusted sub-hazard ratio 0.33, 95% confidence interval 0.11–0.99, *p* = 0.048). The multivariable Cox proportional hazards regression analysis showed that the automated titanium fastener group was not associated with an increased risk of stroke-onset or attaining the composite outcome, respectively, (adjusted hazard ratio 0.54, 95% confidence interval 0.27–1.08, *p* = 0.082), (adjusted hazard ratio 0.65, 95% confidence interval 0.42–1.02, *p* = 0.061). Also, this group was not associated with an increased risk of all-cause mortality, as demonstrated in the multivariable Poisson regression analysis (adjusted incidence-rate ratio 1.42, 95% confidence interval 0.83–2.42, *p* = 0.202).

**Conclusions:**

The use of automated titanium fastener device seems to be safe for infective endocarditis. Analyses of larger cohorts are required.

## Introduction

**Figure 1:**
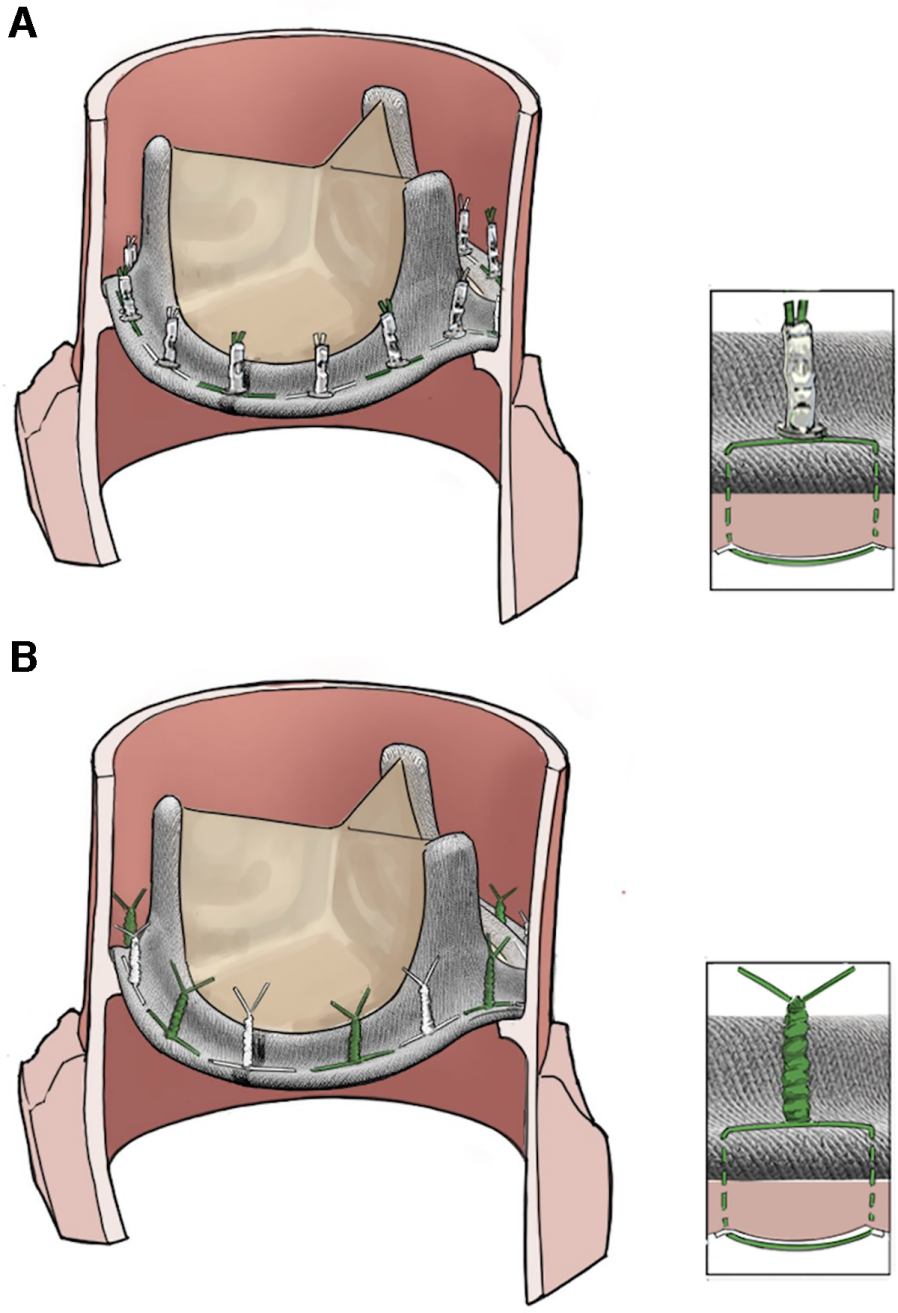
Suture-securing techniques for prosthesis fixation in active IE: (**A**) automated titanium fastener; (**B**) hand-tied knots.

Numerous surgical methods are established for the treatment of active infective endocarditis (IE), including valve replacement with a biological or mechanical prosthesis. For prosthesis fixation, sutures can be secured by either automated titanium fastener (Cor-Knot Device, LSI Solutions, Victor, New York, United States) or conventional hand-tied knots (Figure 1). The automated titanium fastener device enables suture securing with a tiny titanium cylinder. At the suture closure site, the automated titanium fastener is deployed in crimped form, creating a firm suture contact with a single-hand squeeze (1, 2).

Currently, there is no evidence regarding the safety of the automated titanium fastener device in comparison to the hand-tied knots for IE. Indeed, current guidelines do not provide recommendations for the superiority of any suture-securing technique (3, 4).

This study aims to compare clinical outcomes among study groups, based on the suture-securing technique used (automated titanium fastener group vs. hand-tied knots group) for prosthesis fixation in active IE.

## Materials and methods

### Ethical statement

This study was reviewed and approved by the Ethics Committee of the Medical University of Vienna (Ethical Number: 1872/2022; approval date 16.01.2023). The individual patient's consent was waived.

### Patients and clinical data

Patients were screened using the institutional database of the Department of Cardiac Surgery at the Medical University of Vienna. Between January 2016 and December 2022, 220 consecutive patients requiring surgery for active IE were included in this retrospective study. Diagnosis of IE was based on the modified Duke criteria and current guidelines (3–5).

All patients underwent surgical valve replacement with either biological or mechanical prosthesis at the aortic or/and mitral position. The exclusion criteria were age < 18 years, aortic root replacement, homograft implantation, isolated mitral valve repair, and a combination of both suture-securing techniques.

The operations were performed by multiple senior surgeons. For all prostheses, the valve stitch placement technique involved the utilization of U-stitches with pledgets. The surgical treatment of IE at our institution is in line with current guidelines including radical debridement of all infected and necrotic tissue, followed by the reconstruction of any remaining defects (3). Additionally, during the surgery for IE all remaining tissue and surgical pledgets were soaked in an intraoperative disinfectant (povidone-iodine), which is standard practice at our department.

The patients were grouped based on the suture-securing technique used for prosthesis fixation (automated titanium fastener group vs. hand-tied knots group). Operation reports and institutional surgery protocols were reviewed to identify the suture-securing technique. The decision towards using either of the suture-securing techniques was based primarily on the surgeon's personal preference. The use of an automated titanium fastener device started in the year 2016 at our department.

### Study endpoints

The primary study endpoint was re-endocarditis during follow-up. The secondary study endpoints included stroke onset, all-cause mortality, and a composite outcome during follow-up. The composite outcome was defined as an occurrence of either re-endocarditis, stroke, or all-cause mortality during follow-up. A stroke was defined as an acute neurological deficit with evidence of a new lesion on a neuroimaging scan (6). The patient was considered to have had a stroke when either ischemic or hemorrhagic stroke was diagnosed. The study endpoints were identified from patients' medical records. Mortality data were obtained from the Austrian statistics department. No patient was lost to follow-up. A comprehensive dataset was generated through the prospectively maintained database at our department. The last date of follow-up was set for January 5, 2023. The median follow-up time was 664 days for the automated titanium fastener group and 900 days for the hand-tied knots group (*p* = 0.217).

### Statistical analysis

Continuous variables are presented as median and 1st and 3rd quartile, and categorical variables as count and percentages. The Mann–Whitney *U* test was applied to analyze continuous variables with non-normal distributions. For the comparison of categorical variables, the chi-square test was used.

To estimate the effect of the suture-securing technique (automated titanium fastener group vs. hand-tied knots group) on the primary as well as secondary study endpoints, univariable and multivariable proportional hazards regression analyses were performed with the exposure time starting at the date of surgery. Based on directed acyclic graph using current published evidence and clinical experience we selected co-variables to be included in the multivariable models.

The analysis of the primary study endpoint (re-endocarditis) was performed using a multivariable proportional subhazards Fine and Gray regression model accounting for death as a competing event. The primary study endpoint was depicted by the Kaplan–Meier cumulative event curves of both study groups.

The secondary study endpoints: stroke and composite outcome were analyzed using multivariable Cox proportional hazards regression models. The effects were quantified as hazard ratio (HR) with 95% confidence interval (CI). The Kaplan–Meier cumulative event curves for the composite outcome were visualized for both study groups. For the secondary study endpoint, all-cause mortality, the multivariable Poisson regression model was used. The effects were calculated as an incidence-rate ratio with 95% CI.

We used the likelihood ratio test to assess deviations from linearity and to test for interactions.

For superiority, a two-sided *p*-value of less than 0.05 was considered to indicate statistical significance. SPSS 27.0 (IBM Corp, Armonk, NY, USA) and STATA 16.1 software (StataCorp LLC, College Station, TX, USA) were used for statistical analysis.

## Results

### Baseline characteristics

Suture-securing with an automated titanium fastener was performed in 114 (51.8%) patients, whereas the conventional technique of hand knot-tying was used in 106 (48.2%) patients (Table 1). The automated titanium fastener group had a significantly higher rate of preoperative atrial fibrillation (40.4% vs. 27.4%; *p* = 0.042) and diabetes mellitus (27.2% vs. 16.0%; *p* = 0.045). All other baseline characteristics were comparable between groups.

**Table 1:** Baseline characteristics.

Variables	Automated titanium fastener *N* = 114 (51.8%)	Hand-tied knots *N* = 106 (48.2%)	*p*-value
Age (years) (25th–75th interval)	63.4 (52.2–72.5)	61.1 (47.1–69.1)	0.117
Female (%)	32 (28.1)	30 (28.3)	0.970
EuroSCORE II (25th–75th interval)	11.5 (5.2–23.0)	11.3 (5.5–30.3)	0.908
Left ventricular function ≥50% (%)	87 (76.3)	86 (81.1)	0.383
Hypertension (%)	77 (67.5)	63 (59.4)	0.211
Atrial fibrillation (%)	46 (40.4)	29 (27.4)	**0** **.** **042**
Preoperative stroke (within 30 days) (%)	20 (17.5)	26 (24.5)	0.203
Diabetes mellitus (%)	31 (27.2)	17 (16.0)	**0**.**045**
Dialysis (%)	16 (14.0)	14 (13.2)	0.858
Prosthetic valve endocarditis (%)	34 (29.8)	29 (27.4)	0.686
Positive blood culture prior to surgery (%)	28 (24.6)	35 (33.0)	0.166
Negative blood culture after initial evidence of bacteriemia (%)	60 (52.6)	59 (55.7)	0.652
Positive echocardiography findings			
Vegetation (%)	97 (85.1)	92 (86.8)	0.717
Annular abscess (%)	48 (42.1)	38 (35.8)	0.342
Pseudoaneurysm (%)	6 (5.3)	4 (3.8)	0.596
Fistula (%)	2 (1.8)	6 (5.7)	0.122
Peripheral embolism (%)	63 (55.3)	58 (54.7)	0.935
Intravenous drug abuse (%)	11 (9.6)	10 (9.4)	0.957

Bold indicates statistical significance (*p* < 0.05).

Values are presented as *n* (%) or median (interquartile range).

Categorical variables were compared using chi-square test, and continuous variables using Mann–Whitney *U* test.

EuroSCORE II: European System for Cardiac Operative Risk Evaluation II.

### Blood culture results

Table 2 lists species that were identified through blood culture before surgery. The rate of detected species was similar between study groups.

**Table 2:** Blood culture results.

Variables	Automated titanium fastener *N* = 114 (51.8%)	Hand-tied knots *N* = 106 (48.2%)	*p*-value
Staphylococcus aureus (%)	22 (19.3)	23 (21.7)	0.659
Methicillin-resistant Staphylococcus aureus (%)	0 (0.0)	2 (1.9)	0.141
Viridans group streptococci (%)	20 (17.5)	15 (14.2)	0.492
Coagulase-negative staphylococci (%)	12 (10.5)	12 (11.3)	0.850
Enterococcus species (%)	8 (7.0)	13 (12.3)	0.186
Group B Streptococci (%)	2 (1.8)	2 (1.9)	0.941
Enterobacteriaceae (%)	1 (0.9)	3 (2.8)	0.279
Propionibacterium acnes (%)	1 (0.9)	2 (1.9)	0.519
Streptococcus bovis (%)	1 (0.9)	1 (0.9)	0.959
Other (*N* = 1 each) (%)	6 (5.3)	10 (9.4)	0.234
Multiple species (>1 species) (%)	15 (13.2)	11 (10.4)	0.523

Values are presented as *n* (%).

Categorical variables were compared using chi-square test.

### Operative characteristics

The operative characteristics are summarized in Table 3. No differences in eras were observed among study groups. Aortic valve replacement, mitral valve replacement, as well as combined aortic and mitral valve replacement were performed at similar rates. For aortic valve replacement, the Medtronic Avalus prosthesis was implanted significantly more frequently in the automated titanium fastener group (12.3% vs. 4.7%; *p* = 0.046) (Supplementary Table S1). The rate of annular patch augmentation, Commando procedure, ventricular septal defect closure, and aortic root enlargement was similar between groups (Table 3). Coronary artery bypass grafting (CABG) was performed with a significantly higher rate in the automated titanium fastener group (19.3% vs. 9.4%; *p* = 0.038). The cross-clamp times, as well as cardiopulmonary bypass (CPB) times, were comparable among groups.

**Table 3:** Operative characteristics.

Variables	Automated titanium fastener *N* = 114 (51.8%)	Hand-tied knots *N* = 106 (48.2%)	*p*-value
Full sternotomy (%)	111 (97.4)	102 (96.2)	0.630
Re-sternotomy (%)	35 (30.7)	31 (29.2)	0.814
Era (%)			0.091
2016–2019	57 (46.7)	65 (53.3)	
2020–2022	57 (58.2)	41 (41.8)	
Aortic valve replacement (%)	59 (51.8)	55 (51.9)	0.984
Mitral valve replacement (%)	36 (31.6)	30 (28.3)	0.596
Combined aortic and mitral valve replacement (%)	19 (16.7)	21 (19.8)	0.546
Right side involvement	18 (15.8)	9 (8.5)	0.099
Annular patch augmentation (%)	26 (22.8)	29 (27.4)	0.436
Commando procedure (%)	3 (2.6)	3 (2.8)	0.928
Ventricular septal defect closure (%)	1 (0.9)	2 (1.9)	0.519
Aortic root enlargement (%)	7 (6.1)	9 (8.5)	0.502
CABG (%)	22 (19.3)	10 (9.4)	**0** **.** **038**
CPB time (min) (25th–75th interval)	144 (106–192)	145 (105–206)	0.487
Cross clamp time (min) (25th–75th interval)	104 (72–136)	100 (74–137)	0.665

Bold indicates statistical significance (*p* < 0.05).

Values are presented as *n* (%) or median (interquartile range). Categorical variables were compared using chi-square test, and continuous variables using Mann–Whitney *U* test.

CABG, coronary artery bypass grafting; CPB, cardiopulmonary bypass.

### Primary study endpoint

**Figure 2:**
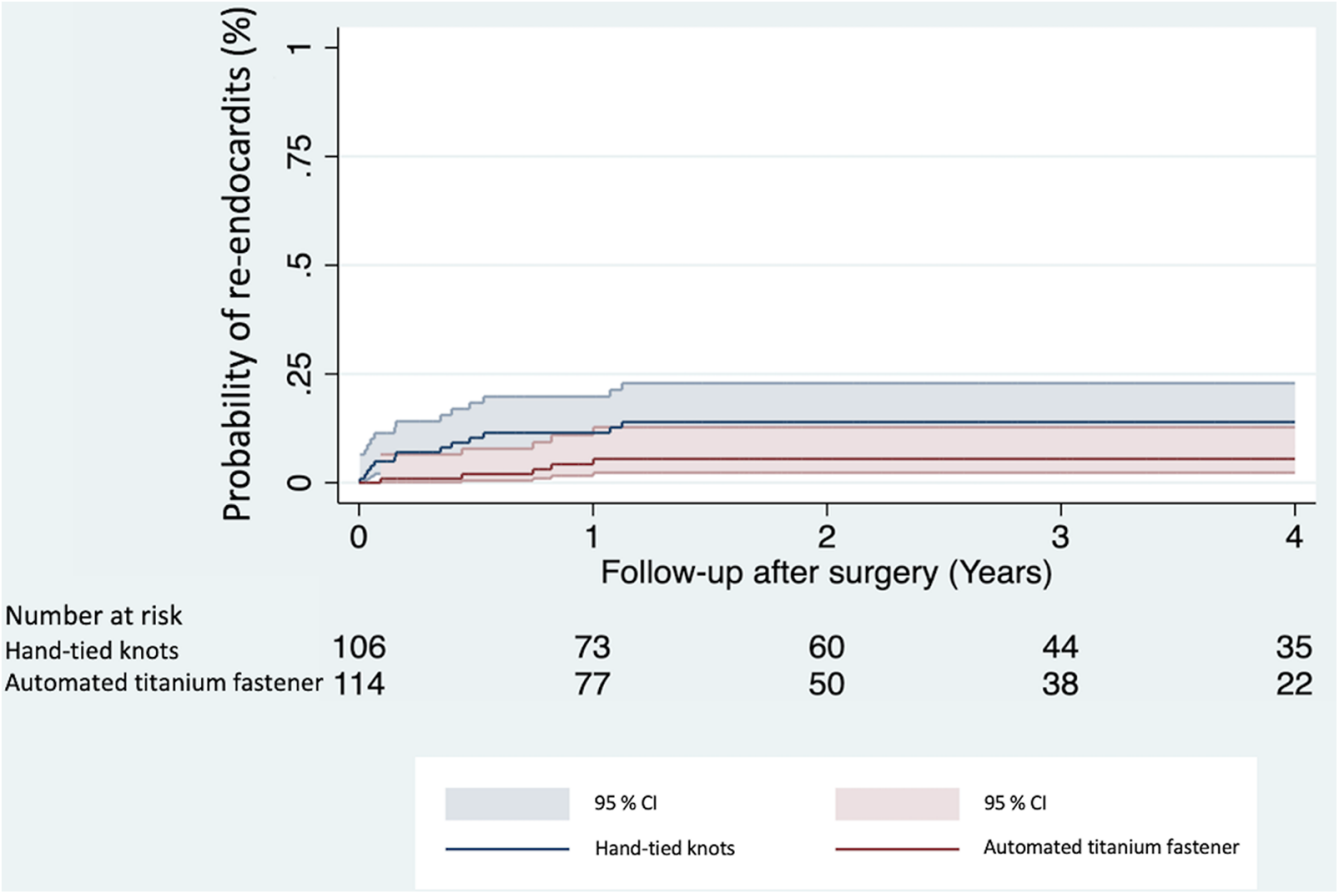
Kaplan-Meier cumulative event curves show the probability of re-endocarditis between the automated titanium fastener and hand-tied knots group. CI, confidence interval.

During the follow-up period, 18 patients (8.2%) reached the primary study endpoint. Univariable Cox proportional hazards regression analysis (Table 4) demonstrated a significantly lower risk of re-endocarditis in the automated titanium fastener group (HR 0.35, 95% CI 0.12–0.96, *p* = 0.042). A multivariable proportional competing risk regression analysis showed that suture-securing with an automated titanium fastener was independently associated with a significantly lower risk of re-endocarditis during follow-up (adjusted sub-hazard ratio 0.33, 95% CI 0.11–0.99, *p* = 0.048) (Table 4; Supplementary Table S2). The median time to re-endocarditis was significantly longer in the automated titanium fastener group (271 days vs. 58 days *p* = 0.040). The Kaplan–Meier cumulative event curves of both study groups are depicted in Figure 2.

**Table 4:** Study endpoints.

	Automated titanium fastener *N* = 114 (51.8%)	Hand-tied knots *N* = 106 (48.2%)	Univariable relative effects		Multivariable relative effects	
	*n* (%)	*n* (%)	95% CI	*p*-value	95% CI	*p*-value
Re-endocarditis^a^	5 (4.4)	13 (12.3)	0.35 (0.12–0.96)	**0** **.** **042**	0.33 (0.11–0.99)	**0**.**048**
Stroke^b^	13 (11.4)	23 (21.7)	0.52 (0.24–1.03)	0.061	0.54 (0.27–1.08)	0.082
All-cause mortality^c^	28 (24.6)	30 (28.3)	1.17 (0.70–1.96)	0.547	1.42 (0.83–2.42)	0.202
Composite outcome^d^	33 (29.0)	49 (46.2)	0.61 (0.39–0.96)	**0**.**031**	0.65 (0.42–1.02)	0.061

Bold indicates statistical significance (*p* < 0.05).

CI, confidence interval; HR, hazard ratio.

^a^ Effects calculated as sub-hazard ratio based on multivariable proportional competing risk regression model.

^b^ Effects calculated as HR based on multivariable Cox proportional hazards regression model.

^c^ Effects calculated as incidence-rate ratio based on multivariable Poisson regression model.

^d^ Effects calculated as HR based on multivariable Cox proportional hazards regression model.

### Secondary study endpoints

During the follow-up period, a stroke occurred in 36 patients (16.4%). The automated titanium fastener was not associated with an increased risk of stroke, as shown in the multivariable Cox proportional hazards regression model (adjusted HR 0.54, 95% CI 0.27–1.08, *p* = 0.082) (Table 4; Supplementary Table S3). Moreover, preoperative stroke (within 30 days) was independently associated with a significantly increased risk of stroke onset during the follow-up period (adjusted HR 2.14, 95% CI 1.02–4.45, *p* = 0.043) (Supplementary Table S3).

Mortality occurred in 58 patients (26.4%) during the follow-up period. As demonstrated in the multivariable Poisson regression analysis, the automated titanium fastener group was not associated with an increased risk of all-cause mortality (adjusted incidence-rate ratio 1.42, 95% CI 0.83–2.42, *p* = 0.202) (Table 4; Supplementary Table S4).

**Figure 3:**
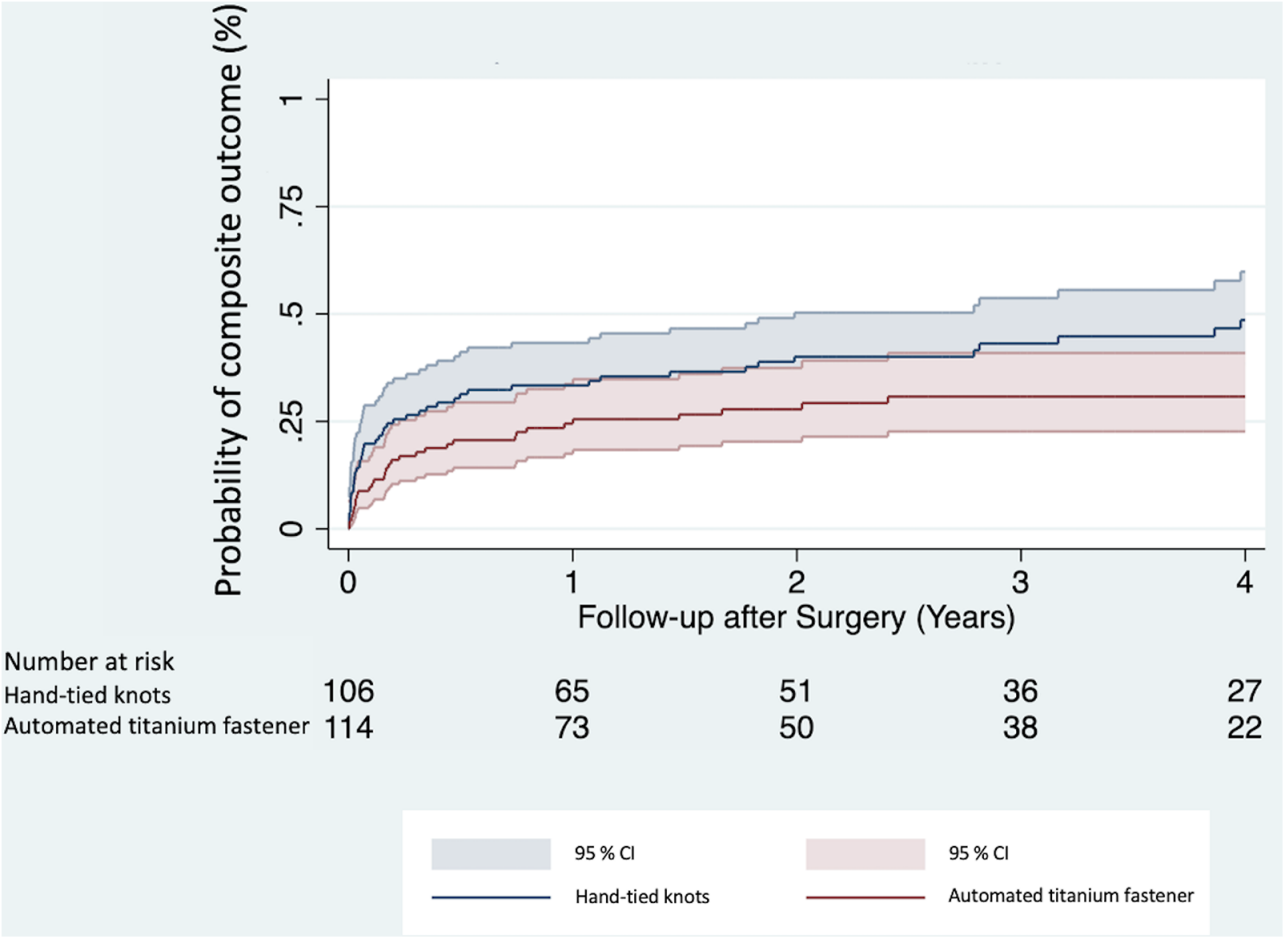
Kaplan–Meier cumulative event curves show the probability of attaining a composite outcome between the automated titanium fastener and hand-tied knots group. CI, confidence interval.

A composite outcome (defined as occurrence of either re-endocarditis, stroke, or all-cause mortality) was reached in 82 (37.3%) patients throughout the follow-up period. Univariable Cox proportional hazards regression analysis (Table 4) showed a significantly lower risk of experiencing the composite outcome in the automated titanium fastener group (HR 0.61, 95% CI 0.39–0.96, *p* = 0.031). In the multivariable Cox proportional hazards regression model, the automated titanium fastener was not associated with an increased risk of attaining the composite outcome (adjusted HR 0.65, 95% CI 0.42–1.02, *p* = 0.061) (Table 4; Supplementary Table S5). The Kaplan–Meier cumulative event curves were visualized for both study groups (Figure 3).

### In-hospital postoperative adverse events and outcomes

The rates of in-hospital postoperative adverse events were similar between the study groups (Table 5). Regarding outcomes, the rate of non-IE-related reoperation was significantly lower in the automated titanium fastener group (0.0% vs. 4.7%; *p* = 0.019) (Table 5). The causes of non-IE-related reoperation in the hand-tied knots group were major paravalvular leak (2.7%), aortic dissection (0.9%), ascending aortic aneurysm (0.9%), and ventricular aneurysm (0.9%). Noteworthy, the rate of re-endocarditis with an initial pathogen was significantly lower in the automated titanium fastener group (1.8% vs. 9.4%, *p* = 0.012).

**Table 5:** In-hospital postoperative adverse events and outcomes.

Variables	Automated titanium fastener *N* = 114 (51.8%)	Hand-tied knots *N* = 106 (48.2%)	*p*-value
In-hospital postoperative adverse events			
Atrial fibrillation (%)	37 (32.5)	36 (34.0)	0.813
Dialysis (%)	21 (18.4)	17 (16.0)	0.640
ECMO support (%)	14 (12.3)	18 (17.0)	0.323
Permanent pacemaker implantation (%)	11 (9.6)	10 (9.4)	0.957
30-day mortality (%)	6 (5.3)	8 (7.5)	0.488
Outcomes			
Stroke			
Ischemic (%)	7 (6.1)	12 (11.3)	0.172
Hemorrhagic (%)	1 (0.9)	5 (4.7)	0.081
Mixed lesion (%)	5 (4.4)	6 (5.7)	0.665
Non-IE related reoperation (%)	0 (0.0)	5 (4.7)	**0**.**019**
Re-endocarditis (%)			
Initial pathogen	2 (1.8%)	10 (9.4)	**0**.**012**
New pathogen	3 (2.6%)	3 (2.8%)	0.928

Bold indicates statistical significance (*p* < 0.05).

Values are presented as *n* (%).

Categorical variables were compared using chi-square test.

### Duration of antibiotic therapy and blood utilization

No significant difference was observed regarding duration of antibiotic therapy between automated titanium fastener group and hand-tied knots group (51 days, interquartile range 42–66, vs. 58 days, interquartile range 43–78; *p* = 0.053) (Table 6).

**Table 6:** Duration of antibiotic therapy and blood utilization.

Variables	Automated titanium fastener *N* = 114 (51.8%)	Hand-tied knots *N* = 106 (48.2%)	*p*-value
Duration of antibiotic therapy			
Duration of antibiotic therapy (days) (25th–75th interval)	51 (42–66)	58 (43–78)	0.053
Blood utilization			
Erythrocyte concentrate (25th–75th interval)	5 (2–8)	5 (3–9)	0.662
Thrombocyte concentrate (25th–75th interval)	1 (0–1)	0 (0–1)	0.627

Values are presented as median and interquartile range.

Continuous variables were compared using Mann–Whitney *U* test.

The administration of erythrocyte concentrate, and thrombocyte concentrate were similar among the study groups during the intraoperative period and throughout the entire postoperative hospital stay, encompassing stay at the intensive care unit, intermediate care unit (if applicable), and standard care unit (Table 6).

### Causes of death

The causes of death were comparable among the study groups (Table 7).

**Table 7:** Causes of death.

Variables	Automated titanium fastener *N* = 114 (51.8%)	Hand-tied knots *N* = 106 (48.2%)	*p*-value
Cardiac (%)	10 (8.8)	10 (9.4)	0.864
Infection (%)	7 (6.1)	11 (10.4)	0.252
Multi-organ failure (%)	6 (5.3)	3 (2.8)	0.363
Neurological (%)	3 (2.6)	4 (3.8)	0.630
Cancer (%)	0 (0.0)	1 (0.9)	0.299
Other (%)	2 (1.8)	1 (0.9)	0.604

Values are presented as *n* (%).

Categorical variables were compared using chi-square test.

## Discussion

The findings derived from the present study are (1): The suture-securing with automated titanium fastener was associated with a significantly lower risk of re-endocarditis during follow-up; (2) The automated titanium fastener group was not associated with an increased risk of stroke, all-cause mortality, or composite outcome during the follow-up; (3) The rate of re-endocarditis with an initial pathogen was significantly lower in the automated titanium fastener group.

To the best of our knowledge, this is the first study to examine the long-term outcomes based on the suture-securing technique used (automated titanium fastener vs. hand-tied knots) during prosthesis fixation in active IE, thereby highlighting its significance and novelty in clinical practice.

Nevertheless, in the elective clinical setting several studies reported the advantages of automated titanium fastener over hand-tied knots in reducing the CPB and cross-clamp time during minimally invasive valve surgery (7, 8). In contrast, we found no differences in either CPB or cross-clamp duration between the study groups, which might be attributed to the variability of IE lesions and the resulting surgical complexity as well as concomitant procedures e.g., CABG surgery.

The automated titanium fastener is designed in a cylindrical shape, composed of surgical titanium. Indeed, a paucity of studies reported that surgical titanium might trigger thrombogenicity, when interfering with blood through the complex series of interconnected processes involving the adhesion and activation of both plasma proteins and platelets and subsequent activation of the intrinsic coagulation pathway (9–12). Of concern, in the context of IE, thrombogenicity is even more pronounced due to the septic condition and ensuing hypercoagulability (12, 13). Interestingly, our analysis shows that the automated titanium fastener was not associated with an increased risk of stroke-onset (adjusted HR 0.54, 95% CI 0.27–1.08, *p* = 0.082). We assume that the flat surface area of the crimped automated titanium fastener causes fewer hemodynamic alterations and therefore might not promote thrombogenicity. Similarly, in elective minimally invasive AVR surgery, Plestis et al. analyzed postoperative clinical outcomes based on the facilitating technologies used (automated titanium fastener and Custodiol solution vs. no-automated titanium fastener and no-Custodiol solution) and found no statistically significant difference in the incidence of stroke among study groups during the hospital stay (14).

The major finding of this study is that suture-securing with an automated titanium fastener is associated with a significantly lower risk of re-endocarditis during follow-up (adjusted sub-hazard ratio 0.33, 95% CI 0.11–0.99, *p* = 0.048). This finding is of particular importance since it might affect surgeon preferences in decision-making towards suture-securing technique for prosthesis fixation in the setting of IE. At this point, no evidence is available addressing this topic. We believe that the flat, smooth surface of the crimped automated titanium fastener around the suture may act as a protective sheath, thus reducing bacterial adhesion. Literature suggests that bacterial adherence and growth are favored on braided sutures, especially on multiple hand-tied knots, thus enhancing the formation of a biofilm (15, 16). The nature of biofilm formation facilitates bacterial resistance to antibiotic treatment (17). Also, the irregular topography of the multiple braided sutures increases the surface area available for bacterial attachment, subsequently increasing the risk of aggravating the infection (16–18).

Furthermore, no-touch principle by using the automated titanium fastener device might be considered as a preferred practice, especially for this subset of patients. It is important to acknowledge that surgeons might inadvertently have contaminated surgical gloves while tying knots manually. Also, in the present analysis, the rate of re-endocarditis with the initial pathogen was significantly lower in the automated titanium fastener group (1.8% vs. 9.4%, *p* = 0.012) (Table 4). Therefore, adhering strictly to the no-touch principle, and minimizing hand contact with the inflamed and infectious environment and with the newly implanted valve prosthesis might be beneficial.

Nonetheless, the cause of re-endocarditis is multifactorial (3). Therefore, this finding should be interpreted with caution.

### Study strengths and limitations

This study represents the first study to analyze long-term clinical outcomes based on the suturing technique used in prosthesis fixation for infective endocarditis, suggesting potentially lower risk of re-endocarditis when utilizing automated titanium fastener device.

Nonetheless, this study had some limitations: (1) It was retrospective single-center study with a relatively small study cohort; (2) Although the study groups were similar, they showed differences in terms of diabetes mellitus, preoperative atrial fibrillation, and CABG—concomitant procedure. (3) The occurrence of re-endocarditis is attributed to multiple factors, irrespective of the suture-securing technique being used; (4) The use of automated titanium fastener was depending on surgeon's preference; (5) The current literature on this topic is sparse.

## Conclusion

Considering the results of the present analysis, the use of automated titanium fastener device seems to be safe in IE and might have potential advantages over the conventional technique of hand knot-tying. Analyses of larger cohorts are required.

## Data Availability

The original contributions presented in the study are included in the article/Supplementary Material, further inquiries can be directed to the corresponding author.
