# Supplementary material for: Automated titanium fastener vs. hand-tied knots for prosthesis fixation in infective endocarditis
Source: Front Cardiovasc Med. 2024 Jan 23;11:1363336. doi: 10.3389/fcvm.2024.1363336 (PMC10844476; doi:10.3389/fcvm.2024.1363336)
Supplement: Supplementary file 1 [file Table1.docx]

Supplementary Material

**Supplemental table S1: Additional procedural data**

| **Variables** | **Automated titanium fastener**  **N =114**  **(51.8%)** | **Hand-tied knots**  **N = 106**  **(48.2 %)** | | | | **p-value** |
| --- | --- | --- | --- | --- | --- | --- |
| Aortic valve replacement - Mechanical (%) | 11 (9.6) | 11 (10.4) | | | | 0.857 |
| Aortic valve replacement - Biological (%) | 48 (42.1) | 44 (41.5) | | | | 0.929 |
| Mitral valve replacement - Mechanical (%) | 11 (9.6) | 10 (9.4) | | | | 0.957 |
| Mitral valve replacement- Biological (%) | 25 (21.9) | 20 (18.9) | | | | 0.574 |
| Combined aortic and mitral valve replacement - Mechanical (%) | 4 (3.5) | 3 (2.8) | | | 0.774 | |
| Combined aortic and mitral valve replacement - Biological (%) | 15 (13.2) | 18 (17.0) | | | 0.427 | |
| Aortic valve prostheses (%) |  |  | | |  | |
| CryoLife On-X Aortic | 14 (12.3) | 14 (13.2) | | | 0.837 | |
| Abbott Masters | 1 (0.9) | 0 (0.0) | | | 0.334 | |
| Edwards Inspiris | 25 (21.9) | 22 (20.8) | | | 0.832 | |
| Edwards Magna Ease | 15 (13.2) | 21 (19.8) | | | 0.183 | |
| Medtronic Avalus | 14 (12.3) | 5 (4.7) | | | **0.046** | |
| Medtronic Mosaic | 6 (5.3) | 8 (7.5) | | | 0.488 | |
| Abbott Trifecta | 1 (0.9) | 3 (2.8) | | | 0.279 | |
| Edwards Intuity | 2 (1.8) | 2 (1.9) | | | 0.941 | |
| Mitroflow Sorin | 0 (0.0) | 1 (0.9) | | | 0.299 | |
| Mitral valve prostheses (%) |  |  | |  | | |
| CryoLife On-X Mitral | 14 (12.3) | | 13 (12.3) | 0.997 | | |
| Abbott Masters | 1 (0.9) | | 0 (0.0) | 0.334 | | |
| Medtronic Mosaic Mitral | 35 (30.7) | | 33 (31.1) | 0.945 | | |
| Edwards Perimount Magna Ease | 5 (4.4) | | 4 (3.8) | 0.819 | | |
| St. Jude Medical Epic | 0 (0.0) | | 1 (0.9) | 0.299 | | |
| Right side involvement | 18 (15.8) | | 9 (8.5) | 0.099 | | |
| Tricuspid valve repair (%) | 13 (11.4) | | 7 (6.6) | 0.216 | | |
| Tricuspid valve replacement (%) | 5 (4.4) | | 2 (1.9) | 0.291 | | |

Bold indicates statistical significance (p < 0.05).

Values are presented as n (%).

Categorical variables were compared using chi-square test.

**Supplemental table S2: Primary study endpoint – Re-endocarditis ^a^**

| **Variables** | **Sub-hazard ratio** | | | **95% CI** | **p-value** |
| --- | --- | --- | --- | --- | --- |
| Automated titanium fastener | | 0.33 | 0.11 – 0.99 | | **0.048** |
| EuroSCORE II (log-transformed) | | 1.10 | 0.62 – 1.93 | | 0.749 |
| Preoperative stroke (within 30 days) | | 2.30 | 0.84 – 6.31 | | 0.105 |
| Prosthetic valve endocarditis | | 2.31 | 0.65 – 8.29 | | 0.198 |
| Intravenous drug abuse | | 2.51 | 0.70 – 8.97 | | 0.158 |
| Annular abscess | | 1.88 | 0.73 – 4.84 | | 0.189 |
| Right side involvement | | 1.10 | 0.21 – 5.48 | | 0.942 |

Bold indicates statistical significance (p < 0.05).

a) Effects calculated as a sub-hazard ratio based on multivariable proportional competing risk regression model.

CI: confidence interval; EuroSCORE II: European System for Cardiac Operative Risk Evaluation II.

**Supplemental table S3: Secondary study endpoint – Stroke ^a^**

| **Variables** | **HR** | **95% CI** | **p-value** |
| --- | --- | --- | --- |
| Automated titanium fastener | 0.54 | 0.27 – 1.08 | 0.082 |
| EuroSCORE II (log-transformed) | 1.41 | 0.95 – 2.13 | 0.091 |
| Age (square root) | 1.00 | 0.99 – 1.00 | 0.530 |
| Atrial fibrillation | 0.89 | 0.42 – 1.89 | 0.754 |
| Preoperative stroke (within 30 days) | 2.14 | 1.02 – 4.45 | **0.043** |
| Diabetes mellitus | 1.16 | 0.50 – 2.73 | 0.728 |
| Dialysis | 1.18 | 0.45 – 3.08 | 0.742 |
| Positive blood culture | 1.39 | 0.47 – 4.10 | 0.548 |
| CPB time | 1.00 | 0.99 – 1.00 | 0.491 |

Bold indicates statistical significance (p < 0.05).

a) Effects calculated as HR based on multivariable Cox proportional hazards regression model.

CI: confidence interval; CPB: cardiopulmonary bypass; EuroSCORE II: European System for Cardiac Operative Risk Evaluation II; HR: hazard ratio.

**Supplemental table S4: Secondary study endpoint – All-cause mortality ^a^**

| **Variables** | **Incidence-rate ratio** | | | **95% CI** | **p-value** |
| --- | --- | --- | --- | --- | --- |
| Automated titanium fastener | | 1.42 | 0.83 – 2.42 | | 0.202 |
| EuroSCORE II (log-transformed) | | 1.87 | 1.36 – 2.58 | | **<0.001** |
| Preoperative stroke (within 30 days) | | 1.87 | 1.02 – 3.42 | | **0.043** |
| Diabetes mellitus | | 1.07 | 0.59 – 1.94 | | 0.818 |
| Positive blood culture | | 2.13 | 0.89 – 5.10 | | 0.088 |
| Right side involvement | | 2.11 | 1.09 – 4.10 | | **0.027** |
| CPB time | | 1.00 | 1.00 – 1.01 | | **0.013** |

Bold indicates statistical significance (p < 0.05).

a) Effects calculated as an incidence-rate ratio based on multivariable Poisson regression model.

CI: confidence interval; CPB: cardiopulmonary bypass; EuroSCORE II: European System for Cardiac Operative Risk Evaluation II.

**Supplemental table S5: Secondary study endpoint – Composite outcome ^a^**

| **Variables** | **HR** | **95% CI** | **p-value** |
| --- | --- | --- | --- |
| Automated titanium fastener | 0.65 | 0.42 – 1.02 | 0.061 |
| EuroSCORE II (log-transformed) | 1.75 | 1.37 – 2.34 | **<0.001** |
| Preoperative stroke (within 30 days) | 1.82 | 1.11 – 2.99 | **0.017** |
| Diabetes mellitus | 1.14 | 0.66 – 1.97 | 0.634 |
| Age (square root) | 0.81 | 0.63 – 1.04 | 0.102 |

Bold indicates statistical significance (p < 0.05).

a) Effects calculated as HR based on multivariable Cox proportional hazards regression model.

CI: confidence interval; EuroSCORE II: European System for Cardiac Operative Risk Evaluation II; HR: hazard ratio.
